# Antifungal Activity of Biosynthesized Silver Nanoparticles (AgNPs) against *Aspergilli* Causing Aspergillosis: Ultrastructure Study

**DOI:** 10.3390/jfb13040242

**Published:** 2022-11-15

**Authors:** Amr H. Hashem, Ebrahim Saied, Basma H. Amin, Fatimah O. Alotibi, Abdulaziz A. Al-Askar, Amr A. Arishi, Fathy M. Elkady, Mostafa A. Elbahnasawy

**Affiliations:** 1 Botany and Microbiology Department, Faculty of Science, Al-Azhar University, Cairo 11884, Egypt; 2 Regional Center for Mycology and Biotechnology, Al-Azhar University, Cairo 11884, Egypt; 3 Department of Botany and Microbiology, Faculty of Science, King Saud University, Riyadh 11451, Saudi Arabia; 4 School of Molecular Sciences, The University of Western Australia, Perth, WA 6009, Australia; 5 Microbiology and Immunology Department, Faculty of Pharmacy (Boys), Al-Azhar University, Cairo 11884, Egypt

**Keywords:** silver nanoparticles, cell-free extract, *Bacillus thuringiensis*, antifungal activity, *Aspergillus*, ultrastructure study

## Abstract

Currently, nanoparticles and nanomaterials are widely used for biomedical applications. In the present study, silver nanoparticles (AgNPs) were successfully biosynthesized using a cell-free extract (CFE) of *Bacillus thuringiensis* MAE 6 through a green and ecofriendly method. The size of the biosynthesized AgNPs was 32.7 nm, and their crystalline nature was confirmed by XRD, according to characterization results. A surface plasmon resonance spectrum of AgNPs was obtained at 420 nm. Nanoparticles were further characterized using DLS and FTIR analyses, which provided information on their size, stability, and functional groups. AgNPs revealed less cytotoxicity against normal Vero cell line [IC_50_ = 155 μg/mL]. Moreover, the biosynthesized AgNPs exhibited promising antifungal activity against four most common *Aspergillus*, including *Aspergillus niger*, *A. terreus*, *A. flavus*, and *A. fumigatus* at concentrations of 500 μg/mL where inhibition zones were 16, 20, 26, and 19 mm, respectively. In addition, MICs of AgNPs against *A. niger*, *A. terreus*, *A. flavus*, and *A. fumigatus* were 125, 62.5, 15.62, and 62.5 μg/mL, respectively. Furthermore, the ultrastructural study confirmed the antifungal effect of AgNPs, where the cell wall’s integrity and homogeneity were lost; the cell membrane had separated from the cell wall and had intruded into the cytoplasm. In conclusion, the biosynthesized AgNPs using a CFE of *B. thuringiensis* can be used as a promising antifungal agent against *Aspergillus* species causing Aspergillosis.

## 1. Introduction

The most common spoilage fungi are *Aspergillus* sp., *Fusarium* sp., *Penicillium* sp., and *Candida* sp.; they can produce mycotoxins, which are extremely harmful to both humans and animals. Symptoms of spoilage fungi can also cause grain discoloration, chemical and nutritional changes, and reduced germination [1,2]. *Aspergillus* species are widespread and their spores contribute up to 22% of the air spore samples [3]. Although there are more than 250 *Aspergillus* species, only a small number of them cause problems in humans [4]. *Aspergillus* infections mostly affect the respiratory system. Inhaled *Aspergillus* spores can germinate in the presence of ideal lung conditions such as high humidity, oxygen, and carbon dioxide [5,6,7].

The most prevalent aspergillosis-causing species is *Aspergillus fumigatus*, followed by *A. flavus* and *A. niger*, while *A. niger* is among the most prevalent infections that cause mycosis in humans. These molds can induce systemic invasive infections and kill at least 50% of infected people [8,9]. In addition to contaminating a variety of nuts, fruits, vegetables, and grains, *A. flavus* also produces aflatoxins, which are mutagenic and carcinogenic [10]. Recently, the emergence of drug-resistant *Aspergillus* species isolates has been reported as a result of prolonged exposure to antifungals [11]. To avoid the generation of more drug-resistant *Aspergillus sps.*, novel antifungal medications are urgently needed for the treatment of aspergillosis. New antifungal agents can be created using nanotechnology to reduce the effects of newly developed resistant fungi [12].

For successful control and treatment of fungal diseases, recent strategies including the production of nanoparticle-based medicines have been addressed [13]. Nanoparticles, such as silver nanoparticles (AgNPs), have been used for a long time as antimicrobial agents and are highly effective against fungal pathogens [14,15,16,17,18]. AgNPs work through a variety of mechanisms, including binding to phosphate groups in DNA [19] and plasma membrane interactions that cause protons to diffuse and cause cell death [20]. They can also interact with sulfhydryl groups in proteins and enzymes, interfere with the electron transport chain, and disrupt membrane permeability to phosphate groups and protons [21]. The production of AgNPs, which makes up almost half of all manufactured nanomaterials globally, is over 450 tons [22]. The commercialization of AgNPs occurs most frequently in the fields of microelectronics, textiles, food and beverage packaging, and healthcare [23]. The traditional methods of chemical synthesis are expensive and result in environmentally hazardous and poisonous chemicals. Therefore, scientists have investigated the ambient biological processes that often take place in the natural systems. The study of natural systems has led to the development of microbial and plant-extract-based methods that have replaced nanosynthesis. Microorganisms have drawn a lot of interest in the green production of nanomaterials [24,25,26,27]. The biological and physicochemical properties of these nanoparticles are valuable in various applications in many fields, including biomedical and agricultural production [13,28,29,30,31,32]. Several microorganisms, including different bacterial species, have successfully been employed for the bio-fabrication of AgNPs [33]. Herein, this study aimed to biosynthesize AgNPs using bacteria and fully characterize them using UV–vis, TEM, FTIR, XRD, and DLS, as well as to study the antifungal activity of AgNPs against aspergilli causing aspergillosis. In addition, ultrastructure changes of highly sensitive *Aspergillus* species were investigated.

## 2. Material and Methods

### 2.1. Isolation of Bacterial Isolates

Twenty bacterial isolates were isolated from different areas. All isolates were screened and tested for the production of AgNPs. The bacterial isolate coded MAE 6 was the only isolate that has a high-yielding capacity for AgNP biosynthesis, which was determined by UV–Vis spectroscopy. This strain was isolated from a soil sample obtained from Egypt’s eastern desert (N: 29°58′49.06″, E: 32°7′3.39″), in a sterilized 250 mL screw-capped bottle. The bacterial strain was isolated using the serial dilution plate method on Luria–Bertani (LB) agar plates. Briefly, 10 mL of sterile water and 1 g of soil sample were combined, well mixed, and serially diluted. On the plates, streaks of the diluted samples were applied. The plates were then incubated at 37 °C for 24 h. A single discrete colony was picked out and re-cultured into fresh plates to check the purity [34].

### 2.2. Identification of Bacterial Strain

#### 2.2.1. Morphological and Biochemical Identification

The isolated pure culture was identified by performing morphological and biochemical tests such as Gram staining, motility, endospore staining, oxidase, catalase, indole, and starch hydrolysis, and glucose fermentation tests [35].

#### 2.2.2. Molecular Identification

The isolated strain was categorized at the species level by 16S rRNA gene sequencing. PCR was employed to amplify the 16S rRNA after the bacterial DNA was separated. This was performed using the 27F and 1492R primers (5’-AGAGTTTGATCCTGGCTCAG-3’ and 5’ GGTTACCTTGTTACGACTT-3’, respectively). A PCR purification kit was used to purify the PCR results, and an ABI 3730xl DNA sequencer (GATC Biotech, Germany) was used to sequence them. In order to compare the amplified product sequences, the BLAST (NCBI) search similarity analysis was used. The strain phylogenetic region was verified by creating a phylogenetic tree using the Neighbor-joining approach using MEGA 7.0 software and 1000 bootstrap repetitions.

### 2.3. Biosynthesis of AgNPs

AgNPs were generated according to a previous protocol [34] by inoculating 200 mL of nutrient broth with the *B. thuringiensis* MAE 6 culture, followed by 24 h of shaking incubation. After incubation, the culture was centrifuged for 20 min at 3075 g to separate the pellet and supernatant. In a clean 250 mL conical flask, supernatant (100 mL) was added, and 1 mL of silver nitrate solution (1 mM) was added. The second reaction served as a control test without AgNO_3_. The mixture was then incubated in a shaking incubator at 200 rpm, 37 °C for 24 h in the dark to prevent oxidation of AgNO_3_. Visual confirmation of AgNP synthesis was investigated by changing the yellow color of the cell-free extract to a brownish color when the precursor was added to it. To create a fine AgNP powder, the supernatant was dried at 60 °C followed by crushing. To completely remove any contaminants from the control flask, AgNPs were thoroughly washed with distilled water and stored for characterization.

### 2.4. AgNP Characterization

AgNPs were characterized according to methods used by Saied et al. [34]. A reaction mix ranging from light yellow to brownish was formed after 24 h. The formation of AgNPs was thought to be indicated by this color change, in which the color shift of control was not seen as compared to the sample. The results were confirmed using conventional characterization methods, such as a UV–visible spectrophotometer (scanning spectra 10^9^ range from 300 to 700 nm, Shimadzu UV-1700, Kyoto, Japan). The morphology of AgNPs was examined using transmission electron microscopy (JEOL 1010 TEM, Japan). The functional groups involved in reducing, capping, and stabilization of AgNPs were studied using Fourier transform infrared spectroscopy (FTIR) (Perkin-Elmer FTIR-1600, USA) by following the standard operating protocol, and the spectral bands of prepared (crystallized form) AgNPs were determined with 400–4000 cm^−1^. X-ray diffraction (XRD) (Shimadzu LabX XRD-6000) with a Cu-Kα X-ray source (λ = 1.5418 Å) analysis was used to evaluate the crystallite nature of AgNPs. The average particle size and size distribution of AgNPs in colloidal solutions were determined by dynamic light scattering (DLS) (The Nicomp ZLS Z 3000, USA).

### 2.5. In Vitro Cytotoxicity of AgNPs against Normal Cell Line

The cytotoxicity of AgNPs and was determined using the MTT protocol [36] with minor modification. An American-type culture collection provided the typical Vero cell line (ATCC). Using the following formula, the total number of cells and the percentage of viable cells were calculated:

$$\mathrm{Viability}\;\%=\frac{\mathrm{Test}\;\mathrm{OD}}{\mathrm{Control}\;\mathrm{OD}}\times 100$$

### 2.6. In Vitro Antifungal Activity of AgNPs

The antifungal activity of AgNPs was evaluated using agar well diffusion against *Aspergillus niger* RCMB 02724, *A. terreus* RCMB 02574, *A. flavus* RCMB 02782, and *A. fumigatus* RCMB 02568. All tested fungal strains were grown on PDA plates and incubated for 3–5 days at 30 °C [37,38]. The fungal suspension was prepared in sterilized phosphate buffer solution (PBS) pH 7.0, and then the inoculum was adjusted to 10^7^ spores/mL after counting in a cell counter chamber. One milliliter was uniformly distributed on agar MEA plates. Using a sterile cork-borer, wells (8 mm) were cut; 100 µL of AgNPs and AgNO_3_ were transferred to each well individually and left for 2 h at 4 °C. Nystatin was used as a standard antifungal, and then the plates were incubated for 3 days at 30 °C. After incubation, the inhibition zones were determined and recorded. Moreover, different concentrations of AgNPs were evaluated as antifungal to detect the minimum inhibitory concentration (MIC).

### 2.7. Ultrastructure Study

An ultrastructure study was carried out on highly AgNPs-sensitive *Aspergillus* sp. to AgNPs. Therefore, this study was performed on *A. terreus* and *A. flavus*. TEM samples were prepared as follows: fungal specimens (nearly 1 mm^3^ each) were removed from agar colonies. The samples were fixed in phosphate buffer, washed in 3% glutaraldehyde, and then post-fixed for five minutes at room temperature in potassium permanganate solution. The samples were dehydrated for 30 min in absolute ethanol after 15 min in each ethanol dilution, which ranged from 10% to 90%. They were subsequently submerged in pure resin after being subjected to a graded series of injections of epoxy resin and acetone. Very thin fragments were collected on copper grids. Sections were then stained three times with uranyl acetate and lead citrate. A JEOL-JEM 1010 transmission electron microscope was employed by the Regional Center for Mycology and Biotechnology (RCMB), Al- Azhar University, to examine stained slices at a voltage of 70 kV [39,40,41].

### 2.8. Statistical Analysis

As all experiments were carried out in triplicate, and the means and standard errors were calculated using SPSS v18 by *t*-test. The significance levels were set at *p* < 0.05.

## 3. Results and Discussion

### 3.1. Isolation and Identification of the Bacterial Isolate Bacillus sp. MAE 6

**Figure 1:**
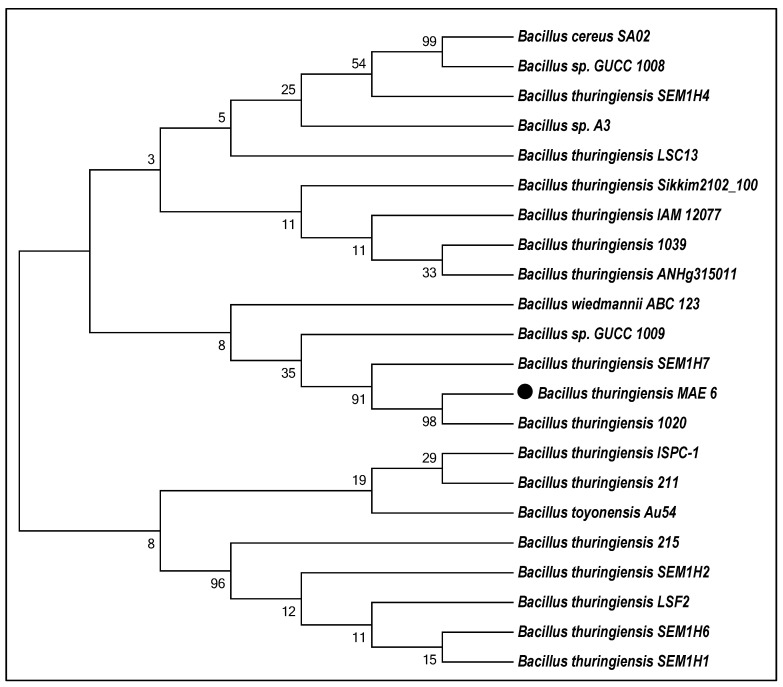
Neighbor-joining phylogenetic tree for *B. thuringiensis* MAE 6 using distance matrix analysis of 16S rRNA gene sequences.

Studying of morphological and biochemical characteristics was carried out to identify the bacterial isolate, and molecular identification (16S rDNA) was performed to confirm the morphological identification. The bacterial isolate MAE 6 was morphologically characterized as Gram-positive endospore formers, and showed negative results for β-xylosidase, d-mannitol, d-mannose, β-glucosidase, and methyl-d-xyloside but positive results for d-glucose, d-trehalose, esculin hydrolysis, phenylalanine arylamidase, and tyrosine arylamidase. The biochemical assays of MAE 6 were carried out by the bioMérieux VITAK2 system and exhibited the identical characteristics of *B. thuringiensis* as described in Bergey’s Manual of Systematic Bacteriology. Supplementary Table S1 displays all of these data. The 16S rDNA fragment of MAE 6 was sequenced for further confirmation of the molecular identity. As illustrated in Figure 1, the MAE 6 phylogenetic tree that was constructed revealed a clustering link between MAE 6 and the *B. thuringiensis* strains. As a result, the bacterial isolate MAE 6 was identified to be *B. thuringiensis* MAE 6, and the sequencing was added to the Gene Bank with the accession number MW547437. The 16S rDNA gene contains highly variable sequences that are a powerful tool in species identification [42]. This technique was used by [42], and they identified bacterial strain KFU36 as *Bacillus* sp. with an identity of 99%. Similarly, Wilson et al. [43] employed the 16S rDNA technique to identify the bacterial isolate (P3) as *B. subtilis* with a similarity of 100%. Moreover, the 16S rDNA shows that the maximum sequence identity (99.48%) of strain AW1-2 was reached with *B. cereus* strain CMS7 (Z2) (KX3443935), *B. anthracis* strain WFS6 (KU977477), and *B. thuringiensis* WG1 (KU977479) [44]. This method was successfully used by Saied et al. [45] to identify HIS7 as a *Lysinibacillus cresolivuorans* strain.

### 3.2. Biosynthesis of AgNPs Using CFE of B. thuringiensis

**Figure 2:**
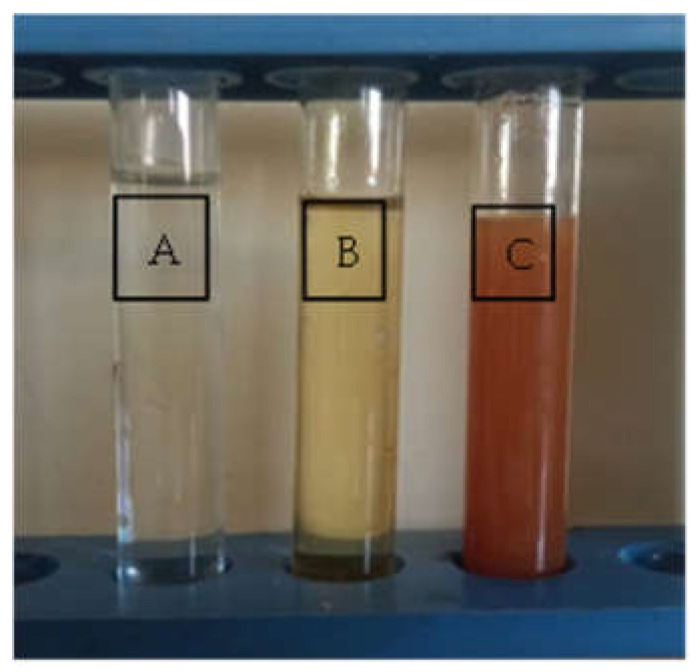
Biosynthesis of AgNPs using *B. thuringiensis* MAE 6. (**A**) Silver nitrate (AgNO3) solution only; (**B**) Cell-free extract (CFE) of *B. thuringiensis* MAE 6; and (**C**) AgNO3+ *B. thuringiensis* MAE 6 CFE showing the brownish color of biosynthesized AgNPs.

The reaction of silver nitrate with the culture supernatant of *B. thuringiensis* MAE 6 and the gradual color change to brown were visually detected after 24 h of incubation, as shown in Figure 2. As a result, the OH groups of the bioactive metabolites in the cell-free extract (CFE) were electrostatically attracted to the positive-charged Ag ions, which reduced to Ag^0^ and eventually led to the creation of AgNPs. The control color did not alter. This result was consistent with those reported for a number of *Bacillus* species, including *B. subtilis* [46], *B. licheniformis* [47], *B. cereus* [48], and *B. thuringiensis* [49]. Liu et al. [50] showed that they synthesized AgNPs by using *Lysinibacillus sphaericus* after 24 h. On the other hand, Nallal et al. [51] succeeded in the biosynthesis of AgNPs by using 1 mM of silver nitrate mixed with an *A. ampeloprasum* bulb extract within 20 min. Additionally, after 24 h of incubation, the green production of AgNPs utilizing *A. sydowii* was accomplished [52].

### 3.3. Characterization of Biosynthesized AgNPs

**Figure 3:**
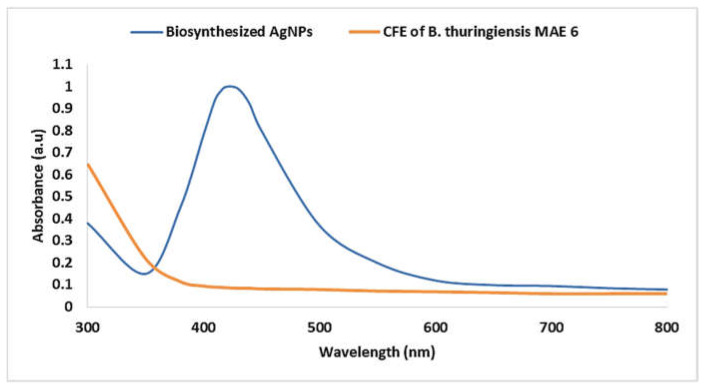
UV–Vis spectra at wavelength 300–800 nm for the biosynthesized AgNPs and CFE of *B. thuringiensis* MAE 6.

A UV–Vis spectrophotometer was used to characterize the prepared biosynthesized AgNPs in the preliminary stage, and it produced an absorbance peak at 420 nm (Figure 3). A powerful localized SPR characteristic was the cause of the NP stimulation. According to the previous explanation, the shape and size of AgNPs are often related to the specific SPR state [53]. Here, AgNPs produced by *B. thuringiensis* MAE 6 had an SPR absorption band at 420 nm. This was accomplished by the free electrons in the AgNPs mutually vibrating with the light wave. SPR values below or higher than 400 nm indicate smaller or bigger nanoparticles, respectively [54]. Similarly, Dawoud et al. [55] reported that the biosynthesis of AgNPs by *Nigrospora oryzae* occurred at an absorbance peak of 420 nm. Additionally, UV–Vis examination of the produced AgNPs revealed a plasmonic peak at 420 nm [56]. Our results on color change and UV–Vis spectral range resembled the findings of Ahmed et al. [57], who reported the color shift of the reaction mixture from yellow to dense brown and the UV–Vis absorption climax of 418.99 nm in the case of AgNPs produced by using the *B. cereus* strain. On the other hand, Nallal et al. [51] proposed the effective and quick synthesis of AgNPs by sunlight using an extract of *A. ampeloprasum*, in which AgNP production was verified by an absorption peak in the UV–Vis spectrum at 446 nm. Using a natural *Bacillus* sp. strain AW1-2, the absorption peak of UV–Vis spectroscopy was detected at 447 nm for the biogenesis of AgNPs [44]. Moreover, Liu et al. [50] reported that the UV−Vis spectra of the biosynthesized AgNPs by using *Lysinibacillus sphaericus* showed a prominent absorbance peak at 413 nm.

#### 3.3.1. XRD Analysis

**Figure 4:**
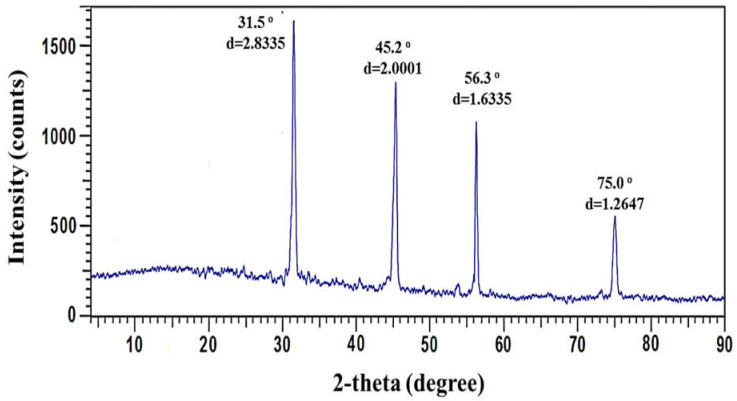
XRD spectrum of biosynthesized AgNPs.

The pure polycrystalline Ag metal was discovered through XRD investigation. AgNPs have been reported to possess a face-centered cubic structure with a comparable crystalline and metallic nature [22,46]. XRD pattern showed the typical Bragg reflections at 2ϴ = 31.5°, 45.2°, 56.3°, and 75.0°, which match the XRD pattern of the indices (111), (200), (220), and (311), as shown in Figure 4. According to the Debye–Scherrer equation, which = 0.9λ/β.cos θ, where 0.1541 nm, the full width at half maximum (FWHM) is the Bragg’s angle in degrees, and D is the average particle size, the average particle size of biogenic AgNPs was determined to be 27.31 nm. The effective production of nanosized particles was proven by the sharp diffraction peak at the lattice plane (111). The XRD measurements obtained agreed with Joint Committee on Powder Diffraction Standards (JCPDS) standard No. 04-0783, confirming that the biosynthesized AgNPs had face-centered cubic (FCC) structures [58,59]. El-Naggar et al. [60] reported the successful fabrication of crystalline AgNPs due to the reduction of Ag ions by phycocyanin, and characterized by face-centered cubic structures. Taran et al. [61] showed that intense peaks corresponding to (111), (200), (220), and (311), which exhibit the formation of AgNPs synthesized by *Bacillus* sp. HAI4, were crystal and face-centered cubic structure in nature. Furthermore, the capping agents may have stabilized NPs, as seen by the strong diffraction peaks seen in the XRD spectra [62]. Unassigned diffraction peaks in the XRD spectra could be caused by biomolecules covering the surface of the crystallizing NPs [63].

#### 3.3.2. FTIR Analysis

**Figure 5:**
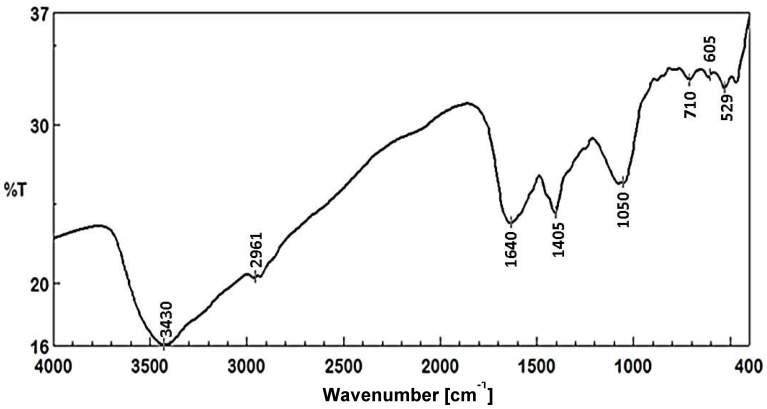
FTIR spectra of biosynthesized AgNPs.

Different biomolecules essential for capping and stabilizing reduced AgNPs as well as reducing silver (Ag^+^) ions were identified through the use of FTIR analysis. Data represented in (Figure 5) revealed intense absorption peaks at 3430, 2961, 1640, 1405, 1050, 710, 605, and 529 cm^−1^. OH and the OH stretching group of phenols and alcohol or NH stretching of aliphatic primary amines might be responsible for the large peak shown at 3430 cm^−1^ [64]. The peak at 2961 cm^–1^ signified C-H vibrational stretching of aliphatic methyl (CH_3_). The peak at 1640 cm^−1^ was associated with I and II amide peptide linkage [65,66]. This amide I band suggests that proteins that may exist in the *B. thuringiensis* MAE 6 cell-free biomass filtrate have the ability to bind to Ag^+^ ions through carboxylate ions or free amine groups to assist in the production of AgNPs during a reduction process. The shifting peak at a wavenumber of the peak at 1405 cm^–1^ can be appointed to C–N stretching vibrations of aliphatic and aromatic amines [67]. The absorption of C-O-C or C-O may be responsible for the peak at 1050 cm^−1^ that was detected [68]. The stretching peaks which appeared at 710 and 605 cm^−1^ indicated the amide IV (OCN) stretch bending for protein and alkene (=C-H bending), respectively. In accordance with other evidence, proteins may serve as capping and/or stability agents during the biosynthesis of AgNPs [69,70]. Elbahnasawy et al. [71] discovered that FTIR spectrum analysis suggested that the cell-free extract of *Rothia endophytica* MAE 11 may function as a reducing and stabilizing agent during the production of AgNPs. Liu et al. [50] showed that FTIR spectra of *Lysinibacillus sphaericus* mediated the synthesis of AgNPs to identify biomolecules involved in the reduction of Ag^+^ ions and capping of biosynthesized AgNPs. Almalki and a Khalifa [42] employed FTIR spectra to identify biomolecules found in the CFE of *Bacillus* sp KFU36, which were used as reducing agents for AgNP synthesis.

#### 3.3.3. DLS and TEM Analyses

**Figure 6:**
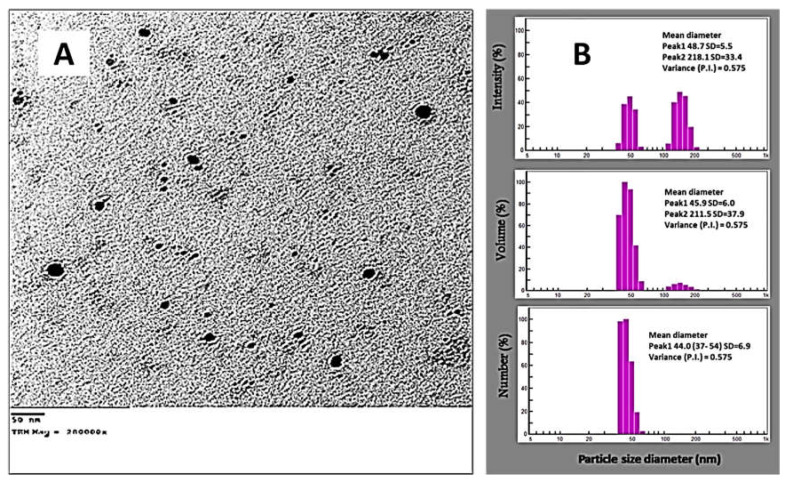
Morphological characterization of biosynthesized AgNPs: (**A**) TEM; (**B**) DLS.

Factors that influence the biotechnological and biological activities of NPs include their size, shape, and distribution [72]. The activity and biocompatibilities of NPs increased as their sizes decreased [63]. Therefore, it is important to examine the NPs’ morphological characteristics. TEM was employed to achieve this objective. Monodispersed spheres were the image of the form of NPs. The size distribution histogram revealed that the average size of the nanoparticles, which ranged in size from 30 to 47 nm, was 32.7 nm (Figure 6A), which was smaller than that formed with *B. thuringiensis* (43.52–142.97 nm) [73]. The results are consistent with other studies that have been published and show that silver oxide nanoparticles produced using *B. linens* varied in size from 25 to 70 nm [74]. Additionally, the size of spherical AgNPs synthesized by *Bacillus* sp KFU36 was extended between 5 and 15 nm [42]. Chi et al. [75] showed that the synthesized BT-AgNPs size ranged from 20.15 to 22.21 nm. Moreover, the AgNPs synthesized by walnut fruit (*Juglans regia*) extract had an average size of 31.4 nm [76]. The TEM image demonstrates that the metabolites in the culture extract are effective in reducing and capping monodispersed spherical AgNPs without aggregation (Figure 6A). Using the DLS method, AgNP size and particle distribution were assessed. The average particle diameter for AgNPs synthesized by *B. thuringiensis* MAE 6 was 37.9 nm for a volume, as illustrated in Figure 6B. The electrical double layer that developed on charged AgNPs and/or coating metabolites on the surface of the nanoparticles utilized for capping and stabilizing AgNPs might be responsible for the DLS’s average size being greater than that generated by TEM [77]. AgNPs synthesized by *B. subtilis* (P3) had an average particle size of 805 nm [43], and this significant disparity could be related to sample variability and synthesis method. A DLS study of silver oxide (Ag_2_O) nanoparticles produced by *B. paramycoides* revealed that the majority of the particles are between 50 and 150 nm in size [74]. According to Saied et al. [34], the average diameter of the AgNPs generated by *Bacillus* sp. was determined to be 55.8 nm for a volume. According to Pavan et al. [78], the DLS examination of the produced AgNPs revealed that the average particle size was 100 nm, and the polydispersity index was 0.224. The average diameter of total particles and stability of green synthesized AgNPs by *S. pachycarpa* extract were reported as 107–150 nm [79]. Nanoparticles with biomolecules attached to their surfaces offer steric stability and stop the particles from aggregation. The osmotic repulsive force improves when the concentration of adsorbed biomolecules increases, because this enhances the stability of nanostructured particles [80,81].

### 3.4. Effect of the AgNPs on the Vero Cells’ Viability Using MTT Assay

**Figure 7:**
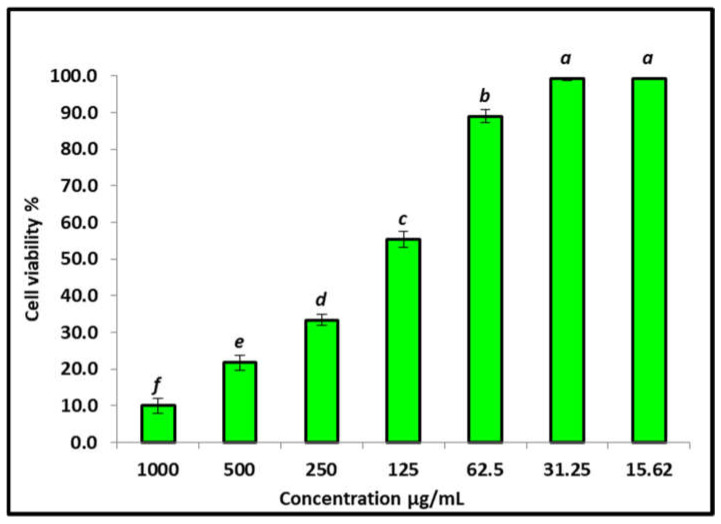
Cytotoxicity of AgNPs against Vero normal cell line (IC_50_ = 155.2 µg/mL). Letters a>b>c>d<e>f mean the power of siginificance.

The in vitro cytotoxic effects of biosynthesized AgNPs were evaluated against a Vero normal cell line and the percentage of cell inhibition was confirmed by MTT assay. The MTT in Vero cells treated with AgNPs at different concentrations (15.62–1000 μg/mL) for 24 h is illustrated in Figure 7. The viability of Vero cells decreased when the AgNPs’ concentration increased. The cell viability was recorded as 99.3% at 15.62 μg/mL of AgNPs. However, cell viability was substantially decreased to 10% at 1000 μg/mL of AgNPs, with a toxicity percent of 0.7% and 90% at 15.62 and 1000 μg/mL, respectively, for Vero cells. A substantial inhibition of Vero cell viability by the AgNPs was detected with an IC_50_ of 155 μg/mL concentrations, indicating cytotoxic effects of these nanoparticles. Similar results agree with data reported by Mohamed et al. [82], who recorded that the normal Vero cell showed 100% viability at 15.62 μg/mL and viability decreased by increasing AgNPs’ concentration: IC50 was recorded at 280 μg/mL. Generally, if the IC50 is ≥ 90 μg/mL, the material is classified as non-cytotoxic [83]. Therefore, the biosynthesized AgNPs in this study are safe for use.

### 3.5. Antifungal Activity

**Figure 8:**
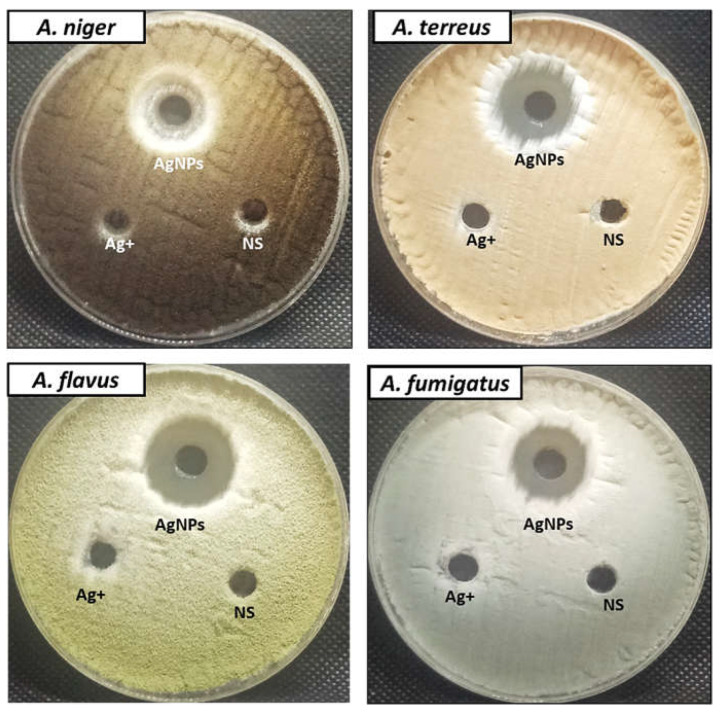
Antifungal activity of AgNPs, AgNO_3_, and Nystatin (NS) at concentration 500 µg/mL against *A. niger*, *A. terreus*, *A. flavus*, and *A. fumigatus* using agar well diffusion method.

In this study, the antifungal activity of AgNPs toward *Aspergillus* species was evaluated using the agar well diffusion method (Figure 8). Results illustrated that biosynthesized AgNPs exhibited promising antifungal activity against all tested fungal strains. AgNPs had the ability to inhibit fungal growth of *A. niger*, *A. terreus*, *A. flavus*, and *A. fumigatus* at concentrations of 500 μg/mL where inhibition zones were 16, 20, 26, and 19 mm, respectively. Moreover, MICs of AgNPs against *A. niger*, *A. terreus*, *A. flavus*, and *A. fumigatus* were determined as illustrated in Table 1 MIC results showed that *A. flavus* was the most sensitive fungus to AgNPs, which inhibited growth at concentrations of 15.6 μg/mL, while for the other fungal species, inhibition of growth was observed at 125 μg/ mL for *A. niger* and 62.5 μg/mL for *A. terreus* and *A. fumigatus*. AgNPs in solution at higher concentrations may be able to adhere to and saturate fungus hyphae, destroying the fungus cells. Such an inhibitory effect can be attributed to Ag^+^ that primarily affects the function of membrane-associated enzymes such as those found in the respiratory chain. Ag^+^ may also affect the expression of some microbial proteins and enzymes. Disruption of DNA replication has also been documented. AgNPs can also interact with substrates through a process known as competitive inhibition, inactivating the enzymes and preventing the production of products required for cell activity. At 50 μg/mL nanoparticle concentrations, the production of AgNPs by *Bacillus* MB353 also exhibited excellent antifungal efficacy against *Aspergillus niger*, *A. fumigatus*, and *F. soleni* [84]. Elbahnasawy et al. [71] reported that the biosynthesized AgNPs using endophytic *Rothia endophytica* have anticandidal activity against *C. albicans*, where the MIC and MBC were 62.5 and 125 μg/mL. The antifungal effectiveness against a well-known phytopathogen *Colletotrichum falcatum* was evaluated by Ajaz et al. [44], where, at a concentration of 20 μg/mL, the ZV-AgNPs greatly inhibited the fungal mycelia in vitro as compared to the untreated control. Particularly, AgNPs prepared by a *Penicillium chrysogenum* and *F. chlamydosporum* cell-free culture filtrates showed significant antifungal efficacy against the mycotoxigenic fungus *A. flavus* and *A. ochraceus* at a concentration of 47–51 μg/mL [85]. Biogenic AgNPs from the extract of *Syzygium cumini* leaves at 100 μg/mL show antifungal activity in both strains *A. flavus* and *A. parasiticus* [86]. At quantities of 50–200 μg/mL, AgNPs produced by *Penicillium verrucosum* inhibited the radial growth of *F. chlamydosporum* and *A. flavus*, reported by [56]. Wang et al. [52] observed AgNPs produced by *A. Sydowii* that exhibited antifungal activity against *A. fumigatus*, *A. flavus*, and *A. terreus*. Dashora et al. [87] observed significant antifungal activity against *A. alternate* at 500 μg/mL of PL-AgNPs mediated by *Polyalthia longifolia* leaf extract.

### 3.6. Ultrastructure Studies by TEM

**Figure 9:**
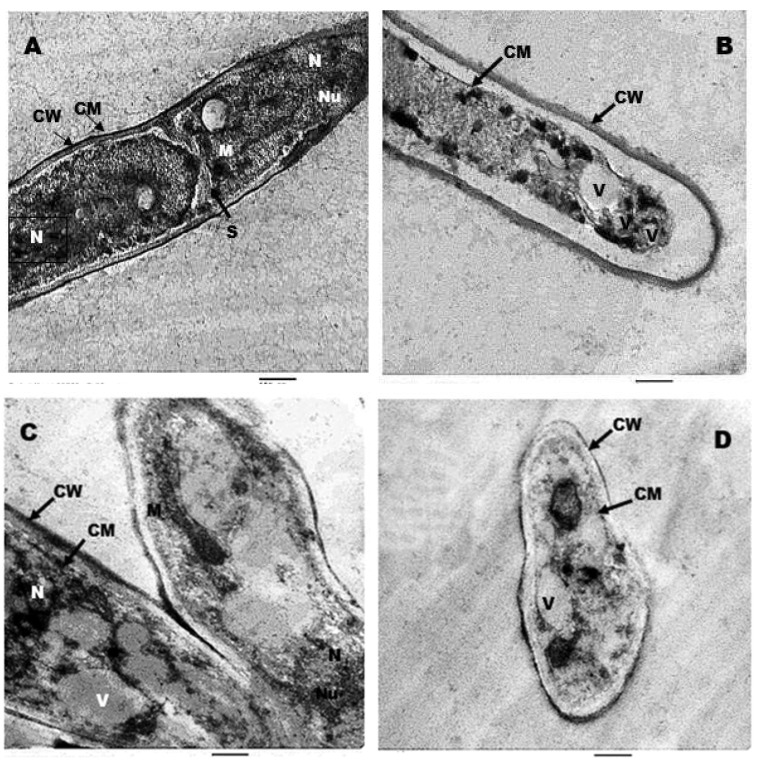
TEM micrographs of *A. terreus* (control (**A**) and treated (**B**)) and *A. flavus* (control (**C**) and treated (**D**)). CW, CM, N, and V mean cell wall, cell membrane, nucleus, and vacuole, respectively. Scale bar = 500 nm.

Ultrathin sections of untreated (control) *A. terrus* (Figure 9A) and *A. flavus* (Figure 9C) cells showed normal mycelia with a consistent layout and homogeneous cytoplasm; the cell wall (CW) of the untreated hypha was fully integrated and undamaged. The cell membrane (CM) was entirely connected to the cell wall and was smooth and wrinkle-free. The cytoplasm was dense and uniform, and the intercellular septum (S) was healthy and unbroken, with normal nucleus (N), nucleolus (Nu), and mitochondria (M) identified. Figure 9B,D show the influence of AgNPs on the cell structure of *A. terrus* and *A. flavus*, respectively. To some extent, the cell wall’s integrity and homogeneity were lost; the cell membrane had separated from the cell wall and had intruded into the cytoplasm. Other modifications included a cytoplasm density decrease and vacuolization (V).

Hyphal filaments in normal samples of *A. terrus* have a classical tubular structure where various organelles, including cell wall and cell membrane, appear as well-established and thick structures. In the treated group, using nanoparticles leads to shrinkage in structure, affecting various organelles and causing enlargement of the volume of vacuoles, which agreed with Al-Otibi et al. [88] who studied ultrastructure alteration in *Bipolaris* sp. upon treatment with AgNPs. Furthermore, photomicrographs of ultrastructure sections of the *A. flavus* normal group revealed the regular cellular organelles including nuclei, cell wall, and cell membrane, while using AgNPs led to decreased cell size and deformation of various organelles. This is in the same line as [89], who reported the beneficial effect of colloidal AgNPs on pathogenic *A. flavus***.** Our results on ultrastructure studies resembled the findings of previous work [90], which imaged the damage to the integrity of the cell wall and cytoplasmic membrane of *Candida albicans* through SEM and TEM studies.

## 4. Conclusions

Green chemistry has been the subject of growing interest due to its being environmentally safe, inexpensive, biocompatible, and that it avoids producing toxic byproducts. In the current study, AgNPs were successfully biosynthesized using the CFE of *B. thuringiensis* MAE 6. According to FTIR analyses, the generated AgNPs are capped and stabilized with various functional groups. The biosynthesized AgNPs showed low cytotoxic activity against Vero normal cell line, with an IC_50_ of 155 μg/mL. Moreover, AgNPs exhibited superior performance in antifungal activities against four types of *Aspergillus* species causing Aspergillosis. Furthermore, an ultrastructure study confirmed the antifungal effect of AgNPs, where the cell wall’s integrity and homogeneity were lost; the cell membrane had separated from the cell wall and had intruded into the cytoplasm. Therefore, the biosynthesized AgNPs using a CFE of *B. thuringiensis* can be used as a promising antifungal agent against *Aspergillus* species.

## Figures and Tables

**Table 1:** Inhibition zones and MICs of AgNPs against *A. niger*, *A. terreus*, *A. flavus*, and *A. fumigatus*.

		AgNPs	AgNO_3_	NS
*A. niger*	IZ/mm	16	ND **	ND
	MIC *	125	ND	ND
*A. terreus*	IZ/mm	20	ND	ND
	MIC	62.5	ND	ND
*A. flavus*	IZ/mm	26	ND	ND
	MIC	15.62	ND	ND
*A. fumigatus*	IZ/mm	19	ND	ND
	MIC	62.5	ND	ND

* MIC by µg/mL ** ND means no activity detected.

## Data Availability

The data used to support the findings of this study are available from the corresponding author upon request.

## Supplementary Materials

The following supporting information can be downloaded at: https://www.mdpi.com/article/10.3390/jfb13040242/s1, Table S1. Characteristics of *B. thuringiensis.*
