# Supplementary material for: Antifungal Activity of Biosynthesized Silver Nanoparticles (AgNPs) against Aspergilli Causing Aspergillosis: Ultrastructure Study
Source: J Funct Biomater. 2022 Nov 15;13(4):242. doi: 10.3390/jfb13040242 (PMC9680418; doi:10.3390/jfb13040242)
Supplement: Supplementary file 1 [file jfb-13-00242-s001.zip › jfb-1998734-supplementary.pdf]

Table S1: Characteristics of *B. thuringiensis.*

Well	Test G+ve endospore	abbreviation	Amount/Well (µg)	6
1	β-xylosidase	BXYL	0.0324	-
3	L-lysine-arylamidase	LysA	0.0228	-
4	L-aspartate arylamidase	AspA	0.024	+
5	leucine-arylamidase	LeuA	0.0234	+
7	phenylalanine arylamidase	PheA	0.0264	+
8	l-proline arylamidase	ProA	0.0234	-
9	β-galactosidase	BGAL	0.036	-
10	L-pyrrolydonyl-arylamidase	PyrA	0.018	+
11	α-galactosidase	AGAL	0.036	-
12	alanine arylamidase	AlaA	0.0222	+
13	tyrosine arylamidase	TyrA	0.0282	+
14	β-n-acetyl-glucosaminidase	BNAG	0.0408	-
15	ala-phe-pro arylamidase	APPA	0.0384	-
18	cyclodextrin	CDEX	0.3	-
19	d-galactose	dGAL	0.3	-
21	glycogen	GLYG	0.1875	-
22	myo-inositol	INO	0.3	-
24	methyl-a-d-glucopyranoside acidification	MdG	0.3	-
25	ellman	ELLM	0.03	-
26	methyl-d-xyloside	MdX	0.3	-
27	α-mannosidase	AMAN	0.036	-

29	maltotriose	MTE	0.3	+
30	glycine arylamidase	GlyA	0.01 2	-
31	d-mannitol	dMAN	0.3	-
32	d-mannose	dMNE	0.3	-
34	d-melezitose	dMLZ	0.3	-
36	n-acetyl-d-glucosamine	NAG	0.3	+
37	palatinose	PLE	0.3	-
39	L-rhamnose	IRHA	0.3	-
41	β-glucosidase	BGLU	0.036	-
43	β-mannosidase	BMAN	0.036	-
44	phosphoryl choline	PHC	0.0366	-
45	pyruvate	PVATE	0.15	+
46	α-glucosidase	AGLU	0.036	-
47	d-tagatose	dTAG	0.3	-
48	d-trehalose	dTRE	0.3	+
50	inulin	INU	0.12	-
53	d-glucose	dGLU	0.3	+
54	d-ribose	dRIB	0.3	+
56	putrescine assimilation	PSCNa	0.201	-
58	growth in 6.5% NaCl	NaCl 6.5%	1.95	+
59	kanamycin resistance	KAN	0.006	+
60	oleandomycin resistance	OLD	0.003	-
61	esculin hydrolysis	ESC	0.0225	+

62	tetrazolium red	TTZ	0.0189	-
63	polymixinj3 resistance	POLYB_R	0.00093	+
Probability (percent)				94% B. thuringiensis
